# Toward Understanding the Dynamics of Microbial Communities in an Estuarine System

**DOI:** 10.1371/journal.pone.0094449

**Published:** 2014-04-14

**Authors:** Weipeng Zhang, Salim Bougouffa, Yong Wang, On On Lee, Jiangke Yang, Colin Chan, Xingyu Song, Pei-Yuan Qian

**Affiliations:** 1 KAUST Global Partnership Program, Division of Life Science, Hong Kong University of Science and Technology, Clear Water Bay, Hong Kong; 2 Sanya Institute of Deep Sea Science and Engineering, Chinese Academy of Sciences, San Ya, Hai Nan, China; 3 CAS/SAFEA International Partnership Program for Creative Research Teams, South China Sea Institute of Oceanology, Chinese Academy of Sciences, China; University of Erlangen-Nuremberg, Germany

## Abstract

Community assembly theories such as species sorting theory provide a framework for understanding the structures and dynamics of local communities. The effect of theoretical mechanisms can vary with the scales of observation and effects of specific environmental factors. Based on 16S rRNA gene tag pyrosequencing, different structures and temporal succession patterns were discovered between the surface sediments and bottom water microbial communities in the Pearl River Estuary (PRE). The microbial communities in the surface sediment samples were more diverse than those in the bottom water samples, and several genera were specific for the water or sediment communities. Moreover, water temperature was identified as the main variable driving community dynamics and the microbial communities in the sediment showed a greater temporal change. We speculate that nutrient-based species sorting and bacterial plasticity to the temperature contribute to the variations observed between sediment and water communities in the PRE. This study provides a more comprehensive understanding of the microbial community structures in a highly dynamic estuarine system and sheds light on the applicability of ecological theoretical mechanisms.

## Introduction

Recent ecological studies have focused on understanding the mechanisms underlying the assembly and dynamics of microbial communities by introducing theoretical frameworks [1]–[5]. For instance, species sorting, that is, the filtering by local environmental conditions is important in assembly of bacterial communities [2], [5]. In addition, adaptive environmental plasticity and the frequency of dispersal can also influence the assembly of microbial communities [6], [7]. These mechanisms have been applied to explain patterns of the distribution, abundance and species interactions of microbiome. However, such patterns can vary with the scale of observation, and different principles might be applicable at different scales.

Compared with microbial surveys in open oceans, lakes, soils, and sewage, studies investigating microbial communities in estuaries are generally lacking. The subtropical Pearl River Estuary (PRE) receives a large volume of nutrient-rich fresh water perennially. In addition, its broad mouth allows seawater from the South China Sea to penetrate the whole estuary, establishing a clear environmental gradient. The environmental conditions in both the water and the sediment have been reported to vary seasonally and spatially [8], [9]. In addition, the Pearl River and its surrounding areas have encountered severe anthropogenic pollution due to the rapid industrialization, increased agricultural activity, and wastewater runoff [10], resulting in highly variable environmental factors in the PRE, such as salinity and nutrient concentration. Considering the variety of natural conditions and human disturbances, the PRE is an ideal site for studying microbial diversity, community structure dynamics, and responses to environmental disturbances, as well as for testing ecological theories.

In the PRE, the surface sediments and their overlying bottom water can be viewed as 2 patches, each containing a community of organisms. These spatially distinct communities are connected to form a metacommunity via the potential dispersal of organisms from one patch to another. However, local conditions from the sediment and water are different from each other and thus, the species sorting and environmental plasticity might be important in shaping the dynamics and structures of communities in this estuarine system. Here, we characterized the surface sediments and their overlying bottom water in the PRE along an environmental gradient in 2 different seasons (June 2009 and January 2010). The analyses were conducted using tagged 16S gene pyrosequencing and analyzed based on metacommunity model predictions. This study provides a more comprehensive understanding of the microbial community structures present in the surface sediment and the overlying bottom water and shed light on the the applicability of ecological theoretical mechanisms.

## Results

### There is a clear delineation between sediment and water community structures

**Figure 1:**
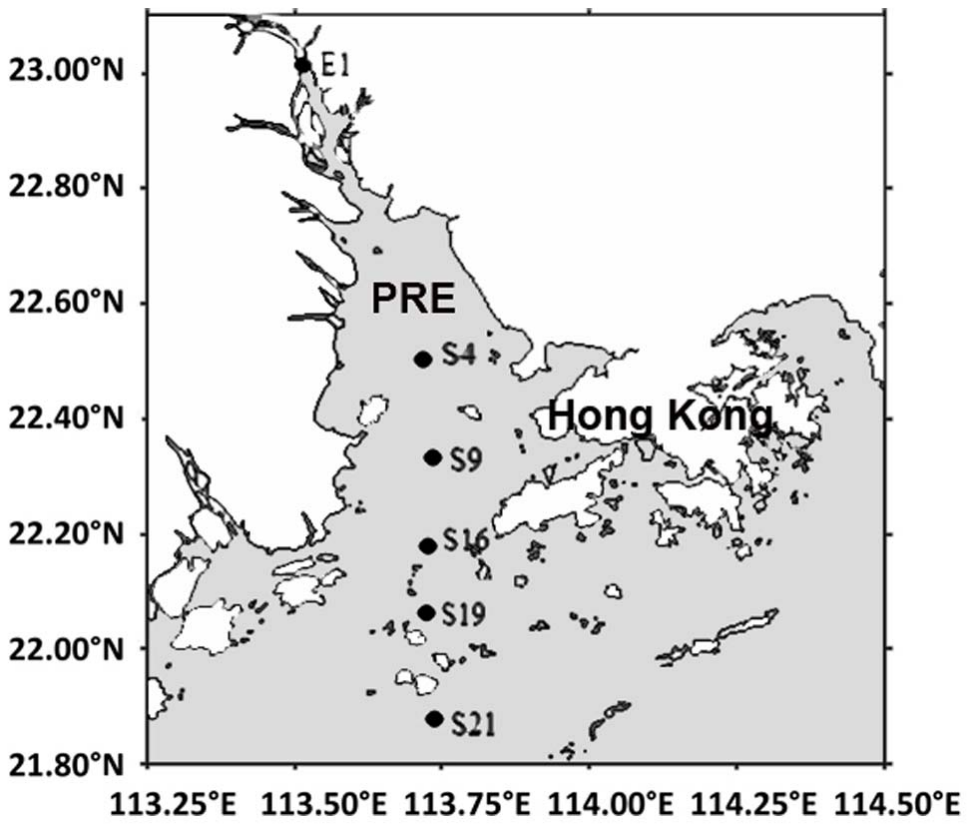
Maps showing the sampling stations. The surface sediment and its overlying bottom water were collected from 6 stations (E1, S4, S9, S16, S19 and S21) along a transect from the Pearl River Estuary (PRE) to the South China Sea.

**Figure 2:**
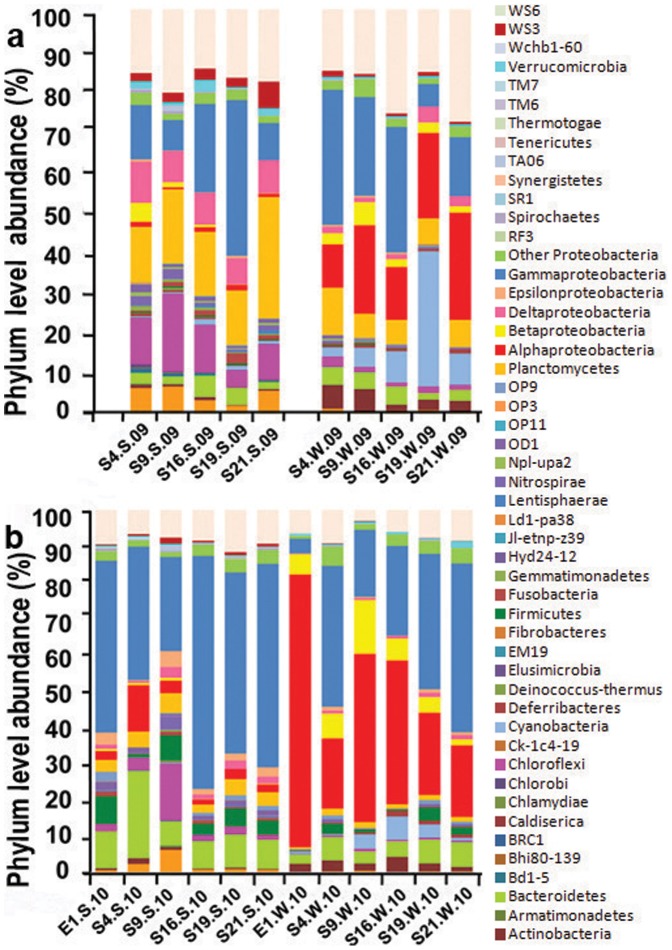
Taxonomic classification of qualified bacterial reads retrieved from samples of the surface sediment and its overlying bottom water at different stations in (a) summer and (b) winter. Qualified reads were assigned to different phyla using the RDP classifier in the QIIME pipeline with a confidence threshold of 80%. “W” represents water samples, “S” represents sediment samples, “09” represents samples collected in the summer of 2009, and “10” represents samples collected in the winter of 2010.

The sampling locations are indicated in figure 1. Regardless of the season, the microbial communities in the surface sediment samples were more diverse than those in the bottom water samples, as indicated by the greater number of OTUs, the Shannon diversity index, and the Chao1 richness estimator (Table 1). For the bacterial reads, 253,493 were assigned to 46 phyla (96,500 from the summer samples and 156,993 from the winter samples), while 36,046 (ie, 12.4%) could not be assigned. The bacterial reads were largely derived from Gammaproteobacteria and Bacteroidetes, which accounted for 5% to 70% of all the samples. However, the proportions of several phyla differed between sediment and water samples. For instance, Alphaproteobacteria was prevalent in the water samples, but its abundance decreased in both the summer and winter sediments. Similarly, Actinobacteria accounted for 2–8% in all the water samples, whereas it diminished in the sediment communities (Fig. 2). Qualified reads were further classified to the genus level, and their relative abundances are displayed in figure S2. More than 600 genera were recovered from the samples, yet only 10 of them were commonly found in all 22 samples. Many of the genera that dominated the microbial communities in the sediments showed a decreased abundance in the bottom water and vice versa.

**Table 1:** Microbial diversity in bottom water and surface sediments from the Pearl River Estuary in summer and winter.

		Summer					Winter				
Sampling station	Sample type	Sample ID	Qualified Reads	OTU	Chao1	Shannon	Sample ID	Qualified Reads	OTU	Chao1	Shannon
E1	Surface sediment	-	-	-	-	-	E1.S.10	17577	1350	2348	7.66
S4	Surface sediment	S4.S.09	7841	2175	3488	10.01	S4.S.10	16728	1082	1698	7.83
S9	Surface sediment	S9.S.09	9596	2094	3193	9.94	S9.S.10	13799	1575	2498	9.1
S16	Surface sediment	S16.S.09	15327	2049	3312	9.56	S16.S.10	13972	1416	2465	7.78
S19	Surface sediment	S19.S.09	12483	1962	3553	8.96	S19.S.10	12991	1584	2680	8.22
S21	Surface sediment	S21.S.09	19708	2239	3627	9.98	S21.S.10	15331	1505	2505	7.84
E1	Bottom water	-	-	-	-	-	E1.W.10	14633	466	845	4.14
S4	Bottom water	S4.W.09	12230	1826	3163	8.72	S4.W.10	25097	1478	2641	7.99
S9	Bottom water	S9.W.09	18071	1613	3092	8.27	S9.W.10	13527	856	1551	6.29
S16	Bottom water	S16.W.09	17401	1408	2607	7.75	S16.W.10	15537	857	1411	6.77
S19	Bottom water	S19.W.09	10190	1165	2194	6.84	S19.W.10	14935	1194	2092	7.70
S21	Bottom water	S21.W.09	11698	1055	1847	7.24	S21.W.10	14953	1350	2255	8.12
			134545					189080			

OTUs were determined at a similarity level of 97%. Values are based on data normalized to the smallest library size (ie, 7841 reads).

**Figure 3:**
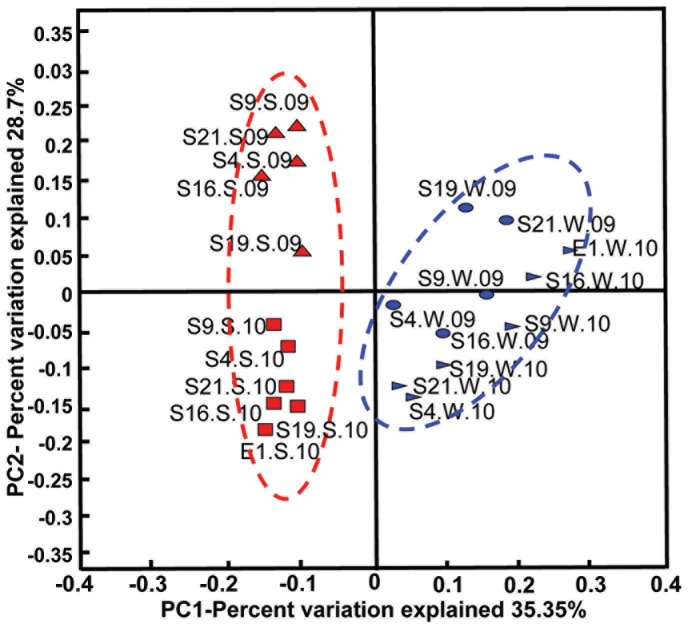
Similarity of microbial communities from the surface sediment and overlying bottom water from the PRE, as illustrated by the UniFrac distance-based PCoA plot. See Table 1 and Figure 2 for the sample identifiers. Ellipses indicate 95% of confidence interval.

Similarity between microbial communities in different samples was supported using jackknife-supported PCoA and PC1 (explaining 35.35% of the variance), which clearly separated microbial communities identified in sediment samples from those in bottom water samples (Fig. 3). PC2 (explaining 28.7% of the variance) further separated the microbial communities identified in summer sediments from those in winter samples. Nonetheless, some of the bottom water samples from both seasons, particularly those from the middle part of the transection, could not be clearly distinguished from one another (Fig.3). These observations were supported by hierarchical jackknife cluster analysis, which showed 2 large clusters with very high bootstrap support for samples from bottom water and from surface sediments (Fig. S3). In addition, a transitional relationship between neighboring communities could be observed for the water samples, as the neighboring communities (S9.W, S16.W, S19.W, S21.W) showed a higher similarity and clustered next to one another (Fig. S3). In contrast, no succession pattern could be observed for the sediment samples. Taken together, these results suggested that the water and sediment community structures could be separated by a delineation based on their structures.

### PRE community dynamics are associated with temperature change

**Figure 4:**
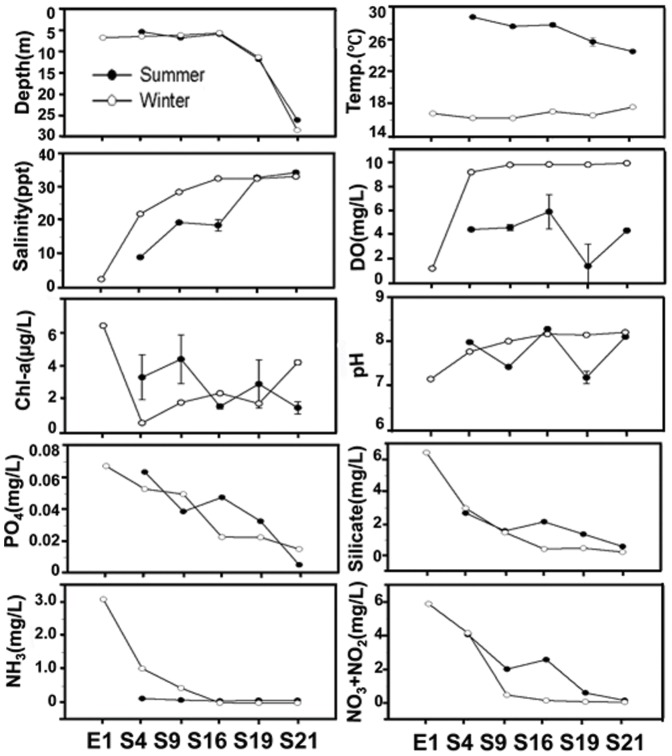
Environmental parameters measured from the overlying water. (a) Depth, (b) salinity, (c) temperature, (d) pH, and (e) dissolved oxygen were measured using a YSI. Nutrients, including (f) PO_4_, (h) SiO_4_, (i) NH_4_, and (j) NO_3_+NO_2_, were measured using a nutrient autoanalyzer. (g) Chlorophyll a (Chl a) content was determined with a fluorometer.

Physicochemical parameters were measured during sampling. As shown in figure 4, the salinity increased along the transect from 9 to 35 ppt in the summer versus 22 to 33 ppt in the winter at S4 to S21, while the lowest value of 2 ppt was recorded at E1. In contrast, NH_3_, NO_3_, NO_2_, silicate, and PO_4_ decreased with increasing sampling depth and salinity and typically displayed slightly higher values in the summer than in the winter at any particular site. The relationships between the microbial community composition and 4 environmental variables: (1) sample type, (2) season, (3) station, and (4) physicochemical parameters measured in different stations were analyzed. The results indicated that both sample type and season on their own could significantly explain up to 31% and 16.5% of the variance in the microbial communities, respectively (Table 2). Among all the physicochemical parameters, the temperature explained the highest percentage of the variance, which collectively explained 14.6% of the total variance. In addition, the station explained the lowest percentage of the variance. These results suggested that seasonal dynamic coupled temperature change might be the main force driving the dynamics of the water and sediment community compositions. To support this conclusion, evidence could be found from the taxonomic profiles in figure 2, as samples from different seasons displayed different patterns. For instance, Deltaproteobacteria, Planctomycetes, Chloroflexi, and Acidobacteria were abundant in the summer sediments but less frequent in the winter sediments. Moreover, it was also suggested by the taxonomic profiles that the microbial communities in the sediment show a greater seasonality than their counterparts located in the water, as Alphaproteobacteria, Betaproteobacteria, Cyanobacteria, and Actinobacteria were common in bottom water samples collected during both seasons (Fig. 2). In addition, One-way ANOSIM was performed to assess the difference between summer water sample from each location and its counterpart in the winter water samples, and the same was done for the sediment groups. As a result, for the water communities, significant differences were only observed between S19.W.09 and S19.W.10 (p<0.01). However, for the sediment samples from all the five locations, significant differences were observed between the summer and winter communities (p<0.01). Consistently, complementary figure 3 showed that the cluster consisting of sediment samples was further separated into 2 subclusters by season, whereas some of the water samples were clustered into one subcluster.

**Table 2:** Summary of RDA.

Factor	Variable	Variance explained	P
Sample type	Sediment	0.310	0.002
	Water	0.310	0.002
Season	Summer	0.165	0.002
	Winter	0.165	0.002
Physiochemical parameters	Temp	0.146	0.012
	DO	0.082	0.124
	Chl-a	0.069	0.174
	NH_3_	0.040	0.550
	PO_4_	0.036	0.562
	NO_3_+NO_2_	0.033	0.674
	Sal	0.031	0.712
	Depth	0.015	0.840
	pH	0.015	0.862
Location	E1	0.031	0.700
	S4	0.028	0.736
	S9	0.039	0.584
	S16	0.012	0.994
	S19	0.022	0.892
	S21	0.020	0.928

RDA was performed for each of the 4 factors (sample type, season, station, and physicochemical parameters) and for all factors to study the variance in the microbial data. Only the first and second axes are shown. Permutation test was performed to assess the significance of the relationship between the environmental factors and the variance.

## Discussion

The key findings of this study are elucidation of the different structures and temperal dynamic patterns of surface sediments and bottom water communities in the PRE, as well as their relationships with environmental factors.

First, PCA analysis indicated delineation between sediment and water community structures at the taxonomic level (Fig. 3) and many genera were specific to sediment or water samples (Fig. S2). These findings suggest that the community structure of water and sediment are shaped by environmental filtering and corelation between the community structure and environmental factors could be observed. For instance, it has been reported that members of the Planctomycetes such as the genera *Candidatus Scalindua, Brocadia*, and *Kuenenia*, which were present in the sediment samples in this study, are capable of anaerobic ammonium oxidation (anammox) in the sediment of some estuarine environments [11]–[15]. Risgaard-Petersen et al [15] reported that the presence of anammox in Randers Fjord estuarine sediment was related to the availability of NO_3_^−^+NO_2_^−^ (NO_x_^−^) in the suboxic zone of the sediment and they confirmed the link between anammox activity to the presence of *Candidatus Scalindua sorokinii*. In this study, we observed an enrichment of *Candidatus Scalindua* and the presence of *Candidatus Brocadia* in the summer sediments, which may be supported by the comparatively higher NO_3_^−^+NO_2_^−^. Therefore, nutrient-based species sorting might be responsible for the different community structures between sediments and water.

Second, the microbial communities in the sediment showed a greater temporal change in the structure than their counterparts located in the water, which is indicative of a higher level of plasticity to temperature of the water communities. A high level of ecological plasticity has been consistently reported for many groups of typical water bacteria [16], [17]. For instance, Polynucleobacter bacteria have been detected in acidic, neutral, and alkaline habitats located in different climatic zones [17]. Moreover, the water samples from both seasons in this study were dominated by Alphaproteobacteria, and several members in this phylum were reported to be temperature adaptive. In contrast, members of *Caldilineales* and *Anaerolinea* in the phylum Chloroflexi were all thermo- or mesophilic and were mostly found in activated sludge samples from waste water treatment plants [18]–[20]. In this study, the Chloroflexi was prevalent in summer sediments but diminished in the winter sediments, and this may explain the effects of temperature on the dynamic of sediment communities.

In addition to nutrient and temperature, previous studies have generally agreed that salinity, could affect microbial density, diversity, and composition in a wide range of aquatic environments [21]–[22]. However, the determinant role of salinity in shaping microbial community composition was not clear in our study. Likely resulting from the high fluctuation of salinity in this complex estuarine environment, the microbes located therein have evolved a high level of plasticity to salinity, and therefore, salinity is not a main driver of the variations in microbial composition. Consistently, several salt-tolerant bacteria, such as members of the *Shewanella, Marinobacter* and *Psychrobacter* [23]–[25] were prevalent in the water and sediment samples in this study. From the theoretical perspective, it could be hypothesized that the environmental factor to which large part of the components of a community have a low plasticity can cause community structure dynamics involving a wide range of taxa.

Finally, other mechanisms, such as dispersal limitation might also be responsible for the different structures and dynamic patterns between the water and sediment communities. Dispersal, namely the movement of organisms across spaces, is a fundamental process that can result in interactions between 2 connected communities [5], [6]. A previous study has reported that although the overlying water and surface sediment are connected, the exchange of particles and nutrients and the movement of water are reduced by a thin sediment–water interface (SWI) [26]. In this study, it is likely to speculate that the SWI produces a physical barrier between the water and sediments in the PRE, limiting the dispersal of bacterial cells. As a result, microbial communities that are specific to the sediment and the water are generated from the different pools of species. However, more evidence based on functional analysis are warranted to support this notion.

In summary, the results presented herein suggest that species sorting by nutrient concentration, temperature change and differences in plasticity dictate the structures and dynamics of microbial communities from the PRE to the South China Sea. However, the mechanism underlying the dynamics of such as metacommunity is complex and may involve many ecological variables that require more empirical approaches and concise measurement techniques such as the determination of dispersal rates. Our future work will focus on functional differences between altered community structures. Moreover, due to difficulties related to the sample collection, we recovered 22 samples from 6 different stations in the PRE. Additional data from a broader range of sites and more replicates are required to validate the present results in surface sediments and bottom water.

## Materials and Methods

### Sample collection and physicochemical parameter measurements

The surface sediment and its overlying bottom water were collected from several stations (E1, S4, S9, S16, S19 and S21) along a transect from the PRE to the South China Sea in June 2009 (summer monsoon and wet season) and January 2010 (winter monsoon and dry season) (Fig. 1). At each sampling station, a suction pump was deployed just above (∼0.5 m) the sea floor, and the whole system was rinsed with *in situ* seawater. After rinsing, 50 L of seawater were collected from the desired depth, of which 12 mL (4 mL each, n = 3) were fixed in 4% formalin to count microbial cells and 1 L was frozen for nutrient analyses in the laboratory. The remainder was immediately filtered through a 1.6-µm GF/A membrane (diameter of 125 mm; Whatman, Clifton, NJ, USA) to remove suspended particles and diatoms, and then through a 0.22-µm Steripak polypropylene filter unit (Millipore, Bedford, MA, USA) to collect the microbial cells for DNA extraction. Twenty-five milliliters of extraction buffer (40 mM EDTA, 0.75 M sucrose, 0.5 M NaCl, 50 mM Tris; pH-8) were then added to the filter unit, and the sample was stored at −80°C until further processing. For the sediment samples, 1 kg of surface sediment was collected using a grab sampler (Van Veen grab, Germany), and then 3 g of surface sediment (1 g each, n = 3) was fixed in 4% formalin for subsequent determination of microbial cell density. The remaining sediment was frozen at −80°C until further use.

The sampling depth varied between 5 and 28 m along the transect from the estuary to the open water. Salinity, temperature, pH, dissolved oxygen, and chlorophyll a (Chl-a) content were measured (5 replicates) on site using a YSI environmental monitoring system (YSI 6600, Yellow Springs Instrument Co., USA) deployed to the depth at which the water was collected. Nutrients, including NO_3_, NH_4_, NO_2_, PO_4_, and SiO_4_, were measured using a Lachat QuickChem 8500 nutrient autoanalyzer (HACH Co., U.S.A) according to standard colorimetric techniques [27]. Large fluctuations in pH and DO were also recorded. Bacterial enumeration was performed following the protocol described previously [28]. Briefly, 5 ml of sterile, distilled water were added in a filtration well. Then 0.5 ml of sample and 25 µl DAPI was added to the filtration well. The filter was placed in a dark area to block any light from hitting the filter. After staining for 3 minutes, a gentle vacuum was applied and the filter (sample side up) was placed onto microscopy slide. Finally, 1 drop of non-fluorescent immersion oil was added on top of the filter before counted on the confocal laser scanning microscopy (LSM7 DUO 710, Carl Zeiss, United States) at 40× magnification. Sediment samples were detached with sonication using 1 s sonication pulses for 30 s. Samples were vortexed for 7 s followed by a shortspin centrifugation for 5 s to settle sediment particles interfering with subsequent processing of samples. The supernatant was transferred into a new Eppendorf tube and used for total cell counting of DAPI stained cells using the same method as bacterial enumeration for water samples.

### Pyrosequencing of barcoded amplicons of the 16S rRNA gene

Upon arrival at the laboratory, total genomic DNA was extracted and purified from the water samples according to the modified SDS-based method described by Lee et al. [29] and by using the PowerSoil DNA Isolation Kit (Mo Bio Laboratories, Inc., Carlsbad, CA, USA). For the sediment samples, DNA was extracted from 10 g of sediment using the PowerMax soil DNA isolation kit (Mo Bio Laboratories, Inc., Carlsbad, CA, USA) according to the manufacturer's protocol. The quality and quantity of the DNA were checked using a NanoDrop spectrophotometer (ND-1000, NanoDrop, USA). Purified DNA samples were maintained at −20°C for future use.

Different samples were PCR-amplified using primers containing an additional 6-nucleotide (nt) barcode (Table S1 in File S1) for multiplexed pyrosequencing. The barcodes were added to the universal forward primer U789F (5′-TAGATACCCSSGTAGTCC-3′) and the reverse primer U1068R (5′-CTGACGRCRGCCATGC-3′) to amplify the hypervariable V6 region of the 16S rRNA genes in bacteria and archaea [30]. The 100-µL PCR reaction mixture contained 5U of *Pfu* Turbo DNA polymerase (Stratagene, La Jolla, CA, USA), 1× *Pfu* reaction buffer, 0.1 mM of each barcoded primer, 0.2 mM of dNTPs (TaKaRa, Dalin, China), and 20 ng of purified DNA template. The PCR was performed in a thermal cycler (Bio-Rad, USA) under the following conditions: initial denaturation at 94°C for 5 min; 26 cycles of 94°C for 30 s, 53°C for 30 s, and 72°C for 45 s; and a final extension at 72°C for 6 min. The PCR products were purified using the TaKaRa Agarose Gel DNA Purification Kit (TaKaRa, China) and quantified using a NanoDrop device. Two mixtures of PCR products were prepared for samples from the 2 different seasons by mixing 200 ng of the purified 16S amplicons from each sample. The samples were then pyrosequenced on a ROCHE 454 FLX Titanium platform (Roche, Basel, Switzerland).

### Calculation of species richness and taxonomic assignment of pyrosequencing reads

The pyrosequencing data were deposited in the NCBI Sequence Read Archive (SRA) database under accession number SRA058403. The downstream bioinformatics analysis was performed using QIIME 1.3.0 [31] with the following quality control criteria: 1) removal of reads with ambiguous nucleotides; 2) removal of reads <150 bp; 3) removal of reads containing homopolymers of ≥6 bp; 4) establishment of a quality window of 50 bp with an average flowgram score of 25. After quality control, 134,545 and 189,080 reads were obtained for the summer and winter samples, respectively. The reads were assigned to their corresponding samples according to their barcodes, denoised using Denoiser [32], clustered using uclust [33], and then assigned to operational taxonomic units (OTUs) at 3% dissimilarity. The most abundant reads were selected as representatives from each OTU for *de novo* alignment using MUSCLE [34 and alignment against the Silva108 database using PyNAST [35]. Among the reads that were aligned successfully, chimeras were identified using ChimeraSlayer [36] and then removed from the dataset. The species diversity, richness, and rarefaction curves (Fig. S1) were computed at 3% dissimilarity as part of the QIIME alpha diversity pipeline, while the beta diversity was analyzed after rarefying of the samples in the smallest-sized library using a step size of 100 with 100 repetitions per step. The reads were assigned to different taxa using the RDP classifier version 2.2 [37] against Silva108 [38] with a bootstrap confidence level of 80%, and 97% of the OTUs recovered from the samples were assigned to Bacteria and the remainder to Archaea (Table 1 and Table S2 in File S1).

### Comparison of microbial communities and their relationship with the environment

The similarities among different microbial communities were determined by similarity matrices generated based on the phylogenetic distance between reads (ie, Unifrac distance; [39]) and displayed using jackknifed principle coordinate analysis (PCoA) and the unweighted pair group method with arithmetic mean (UPGMA) clustering implemented in the QIIME pipeline. We used the ellipse option in SYSTAT [40] to estimate 95% confidence ellipses for the principal component scores of water and sediment samples separately. In addition, the numbers of reads assigned to different genera were converted into percentages, which served as an input for Cluster3 [41]. Genera with a low relative abundance of less than 0.02% of all 22 samples were removed. The remaining genera were further normalized and centered by the mean. The complete linkage method with a metric of correlation (uncentered) was used to generate a hierarchical cluster and a heat map using Java TreeView [42].

The correlations between microbial assemblages with sample type (sediment vs water), season (summer vs winter), and the measured physicochemical parameters among different stations were analyzed using ordination methods with the software Canoco (version 4.5, Microcomputer Power, USA). For both constrained and unconstrained ordination methods, the percent abundance data for the microbial groups (at the genus level) in each library were used as the ‘species data’, and the environmental variables, normalized by log transformation, served as the ‘environmental data’. Significance was assessed for the first axis and for all canonical axes using 499 Monte-Carlo permutations under a reduced model. Where appropriate, forward selection was used to build optimal models for the microbe-environment relationship. One-way ANOSIM test in the PAST [43] software package was used to test the difference between bacterial community structures based ob the OTU abundance using a P value of 0.01.

## Supporting Information

Figure S1 Rarefaction curves for microbial communities in the surface sediment and overlying water from the Pearl River Estuary from (a) summer and (b) winter. Rarefaction is shown for OTUs at a dissimilarity level of 3%. See Table 1 and Figure 2 for the sample identifiers.
(TIF)

Figure S2 Heat map showing the relative abundance and distribution of representative 16S rRNA tagged sequences classified at the genus level. The normalized data were clustered using the complete linkage method and a metric of correlation (uncentered). The color code indicates the difference in relative abundance from the mean, ranging from green (-ve), to black (mean) and to red (+ve). See Table 1 and Figure 2 for the sample identifiers.
(TIF)

Figure S3 Similarity of microbial communities from the surface sediment and overlying bottom water from the Pearl River Estuary, as illustrated by UPGMA jackknifed hierarchical clustering. Bootstrap values larger than 50% of the 1,000 resamplings are shown at the nodes. See Table 1 and Figure 2 for the sample identifiers.
(TIF)

File S1 Contains Table S1, barcoded primers for obtaining 16S rDNA amplicons from the bottom water and surface sediment samples. Table S2, microbial diversity in the bottom water and surface sediments from the Pearl River Estuary in summer and winter.
(DOC)
