# Supplementary figures and images for: Toward Understanding the Dynamics of Microbial Communities in an Estuarine System
Source: PLoS One. 2014 Apr 14;9(4):e94449. doi: 10.1371/journal.pone.0094449 (PMC3986090; doi:10.1371/journal.pone.0094449)

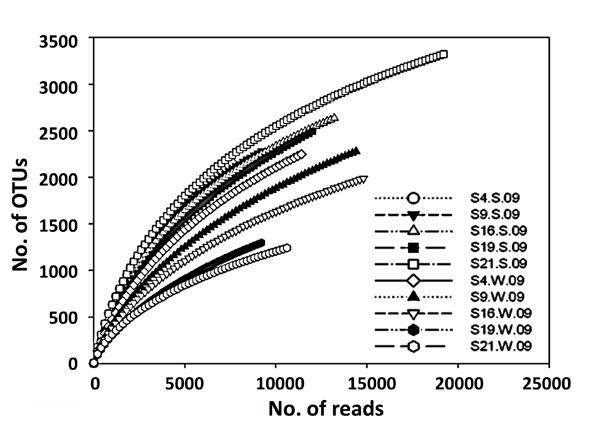

Supplement: Figure S1 — Rarefaction curves for microbial communities in the surface sediment and overlying water from the Pearl River Estuary from (a) summer and (b) winter. Rarefaction is shown for OTUs at a dissimilarity level of 3%. See Table 1 and Figure 2 for the sample identifiers. (TIF) [file pone.0094449.s001.tif]

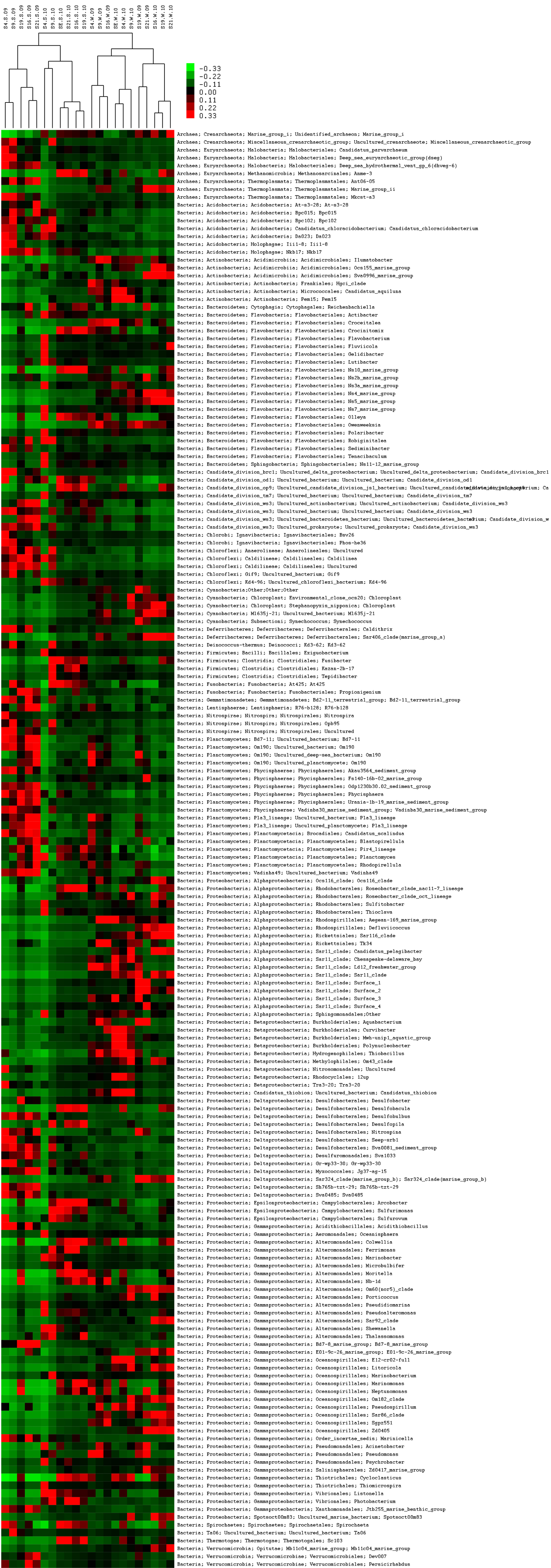

Supplement: Figure S2 — Heat map showing the relative abundance and distribution of representative 16S rRNA tagged sequences classified at the genus level. The normalized data were clustered using the complete linkage method and a metric of correlation (uncentered). The color code indicates the difference in relative abundance from the mean, ranging from green (-ve), to black (mean) and to red (+ve). See Table 1 and Figure 2 for the sample identifiers. (TIF) [file pone.0094449.s002.tif]

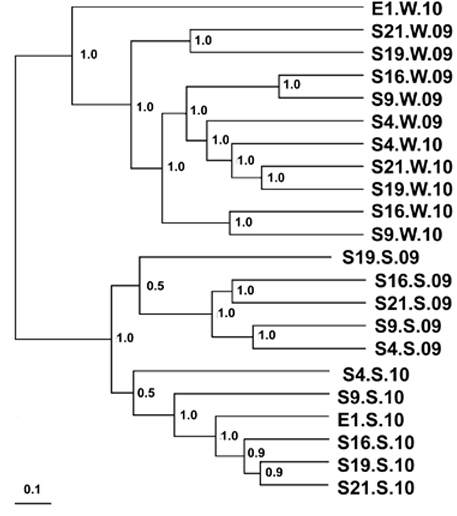

Supplement: Figure S3 — Similarity of microbial communities from the surface sediment and overlying bottom water from the Pearl River Estuary, as illustrated by UPGMA jackknifed hierarchical clustering. Bootstrap values larger than 50% of the 1,000 resamplings are shown at the nodes. See Table 1 and Figure 2 for the sample identifiers. (TIF) [file pone.0094449.s003.tif]
