# Supplementary material for: Toward Understanding the Dynamics of Microbial Communities in an Estuarine System
Source: PLoS One. 2014 Apr 14;9(4):e94449. doi: 10.1371/journal.pone.0094449 (PMC3986090; doi:10.1371/journal.pone.0094449)
Supplement: File S1 — Contains Table S1, barcoded primers for obtaining 16S rDNA amplicons from the bottom water and surface sediment samples. Table S2, microbial diversity in the bottom water and surface sediments from the Pearl River Estuary in summer and winter. (DOC) [file pone.0094449.s004.doc]

Table S1. Barcoded primers for obtaining 16S rDNA amplicons from the bottom water and surface sediment samples. A 6-nucleotide barcodes was designed and attached to the universal primers (U789F: 5’- TAGATACCCSSGTAGTCC-3’; U1068R: 5’- CTGACGRCRGCCATGC-3’) for amplification of the 16S rDNA from different samples. Amplicons from the summer and winter samples were pyrosequenced in two separated runs.

| Sample ID | Location | Type | Season | Barcode |
| --- | --- | --- | --- | --- |
| S4.S.09 | S4 | Surface sediment | Summer | CTATAT |
| S9.S.09 | S9 | Surface sediment | Summer | CTATCA |
| S16.S.09 | S16 | Surface sediment | Summer | CTACTG |
| S19.S.09 | S19 | Surface sediment | Summer | CTACAC |
| S21.S.09 | S21 | Surface sediment | Summer | CTAGCG |
| S4.W.09 | S4 | Bottom water | Summer | TGATAT |
| S9.W.09 | S9 | Bottom water | Summer | TGACAC |
| S16.W.09 | S16 | Bottom water | Summer | TGAGCG |
| S19.W.09 | S19 | Bottom water | Summer | ACATAT |
| S21.W.09 | S21 | Bottom water | Summer | ACATCA |
| E1.S.10 | E1 | Surface sediment | Winter | CTATAT |
| S4.S.10 | S4 | Surface sediment | Winter | CTATCA |
| S9.S.10 | S9 | Surface sediment | Winter | CTACTG |
| S16.S.10 | S16 | Surface sediment | Winter | CTACAC |
| S19.S.10 | S19 | Surface sediment | Winter | CTAGCG |
| S21.S.10 | S21 | Surface sediment | Winter | TGATCA |
| E1.W.10 | E1 | Bottom water | Winter | TGACTG |
| S4.W.10 | S4 | Bottom water | Winter | TGACAC |
| S9.W.10 | S9 | Bottom water | Winter | ACATAT |
| S16.W.10 | S16 | Bottom water | Winter | ACATCA |
| S19.W.10 | S19 | Bottom water | Winter | ACACTG |
| S21.W.10 | S21 | Bottom water | Winter | ACACAC |

Table S2 Microbial diversity in the bottom water and surface sediments from the Pearl River Estuary in summer and winter. OTUs were determined at a similarity level of 97%. Values are based on non-normalized data.

| Sample ID | Qualified reads | Unclassified reads | Bacterial reads | Archaeal reads | Bacterial OTUs | Archaeal OTUs | OTUs |
| --- | --- | --- | --- | --- | --- | --- | --- |
| S4.S.09 | 7841 | 102 | 7406 | 333 | 2063 | 112 | 2175 |
| S9.S.09 | 9596 | 192 | 8883 | 521 | 2162 | 140 | 2302 |
| S16.S.09 | 13527 | 2003 | 13011 | 313 | 2543 | 101 | 2644 |
| S19.S.09 | 12483 | 334 | 11948 | 201 | 2435 | 72 | 2507 |
| S21.S.09 | 19708 | 426 | 18507 | 775 | 3204 | 121 | 3325 |
| S4.W.09 | 12230 | 801 | 11107 | 322 | 2204 | 45 | 2249 |
| S9.W.09 | 18071 | 3607 | 14067 | 397 | 2235 | 48 | 2283 |
| S16.W.09 | 17401 | 2429 | 14656 | 316 | 1950 | 47 | 1997 |
| S19.W.09 | 10190 | 931 | 9084 | 175 | 1268 | 32 | 1300 |
| S21.W.09 | 11698 | 999 | 10069 | 630 | 1199 | 47 | 1246 |
| E1.S.10 | 17577 | 2173 | 15248 | 156 | 1904 | 40 | 1944 |
| S4.S.10 | 16728 | 660 | 15988 | 80 | 1445 | 26 | 1471 |
| S9.S.10 | 13799 | 240 | 13283 | 276 | 1908 | 81 | 1989 |
| S16.S.10 | 13972 | 613 | 13299 | 60 | 1857 | 16 | 1873 |
| S19.S.10 | 12991 | 851 | 12035 | 105 | 1971 | 35 | 2006 |
| S21.S.10 | 15331 | 868 | 14346 | 117 | 1997 | 27 | 2024 |
| E1.W.10 | 14634 | 1957 | 12652 | 24 | 611 | 17 | 628 |
| S4.W.10 | 25097 | 2185 | 22813 | 99 | 2429 | 31 | 2460 |
| S9.W.10 | 13527 | 2065 | 11372 | 90 | 1088 | 27 | 1115 |
| S16.W.10 | 15537 | 2128 | 12966 | 443 | 1119 | 35 | 1154 |
| S19.W.10 | 14935 | 1282 | 13356 | 297 | 1575 | 41 | 1616 |
| S21.W.10 | 14953 | 1078 | 13443 | 432 | 1764 | 42 | 1806 |
